# A second monoclinic modification of triphenyl­phosphine oxide hemihydrate

**DOI:** 10.1107/S1600536809019254

**Published:** 2009-05-29

**Authors:** Seik Weng Ng

**Affiliations:** a Department of Chemistry, University of Malaya, 50603 Kuala Lumpur, Malaysia

## Abstract

In the crystal of the title compound, C_18_H_15_OP·0.5H_2_O, a water molecule links to two adjacent triphenylphosphine molecules by way of O—H⋯O hydrogen bonds. The crystal is twinned, the minor twin component being 36%.

## Related literature

For the *C*2/*c* modification, see: Baures (1991 ▶). For the *Fdd*2 modification, see: Baures & Silverton (1990 ▶) (the authors mention a *Cc* modification without providing details).
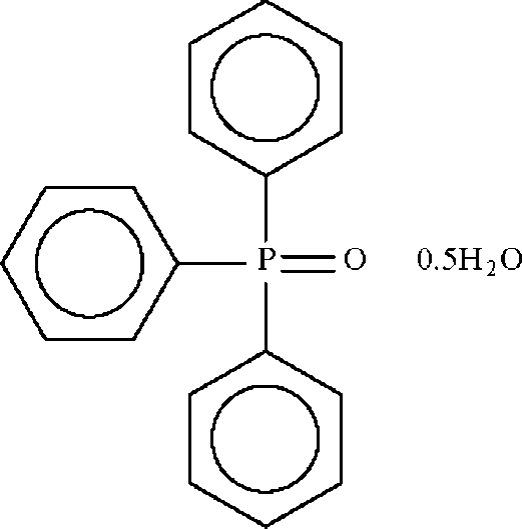


## Experimental

### 

#### Crystal data

C_18_H_15_OP·0.5H_2_O
*M* *_r_* = 287.28
Monoclinic, 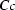

*a* = 9.4313 (1) Å
*b* = 32.1930 (4) Å
*c* = 10.8435 (1) Å
β = 115.742 (1)°
*V* = 2965.59 (6) Å^3^
*Z* = 8
Mo *K*α radiation
μ = 0.18 mm^−1^
*T* = 100 K
0.30 × 0.25 × 0.20 mm

#### Data collection

Bruker SMART APEX diffractometer
Absorption correction: multi-scan (*SADABS*; Sheldrick, 1996 ▶) *T*_min_ = 0.947, *T*_max_ = 0.965
14206 measured reflections
6609 independent reflections
6504 reflections with *I* > 2σ(*I*)
*R*_int_ = 0.019

#### Refinement

*R*[*F* ^2^ > 2σ(*F* ^2^)] = 0.026
*wR*(*F* ^2^) = 0.065
*S* = 1.02
6609 reflections
379 parameters
4 restraints
H atoms treated by a mixture of independent and constrained refinement
Δρ_max_ = 0.24 e Å^−3^
Δρ_min_ = −0.17 e Å^−3^
Absolute structure: Flack (1983 ▶), 3203 Friedel pairs
Flack parameter: 0.02 (5)

### 

Data collection: *APEX2* (Bruker, 2008 ▶); cell refinement: *SAINT* (Bruker, 2008 ▶); data reduction: *SAINT*; program(s) used to solve structure: *SHELXS97* (Sheldrick, 2008 ▶); program(s) used to refine structure: *SHELXL97* (Sheldrick, 2008 ▶); molecular graphics: *X-SEED* (Barbour, 2001 ▶); software used to prepare material for publication: *publCIF* (Westrip, 2009 ▶).

## Supplementary Material

Crystal structure: contains datablocks I, global. DOI: 10.1107/S1600536809019254/xu2526sup1.cif

Structure factors: contains datablocks I. DOI: 10.1107/S1600536809019254/xu2526Isup2.hkl

Additional supplementary materials:  crystallographic information; 3D view; checkCIF report

## Figures and Tables

**Table 1:** Hydrogen-bond geometry (Å, °)

*D*—H⋯*A*	*D*—H	H⋯*A*	*D*⋯*A*	*D*—H⋯*A*
O1*W*—H1⋯O1	0.84 (3)	2.05 (2)	2.836 (2)	156 (4)
O1*W*—H2⋯O2	0.83 (3)	2.09 (2)	2.870 (2)	158 (3)

**Fig. 1.**
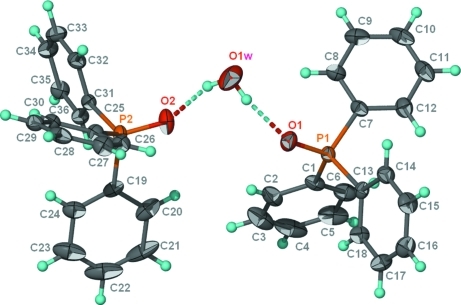
The molecular structure of the title compound. Displacement ellipsoids are drawn at the 70% probability level, and hydrogen atoms are drawn as spheres of arbitrary radius. Dashed lines denote hydrogen bonds.

## supplementary crystallographic information

### Experimental

The compound was the unexpected crystalline product of the reaction between tetracyclohexyltin (1 g, 2.2 mmol) and dibromotriphenylphosphine (1 g, 2.4 mmol) in ethanol. The reactants were heated in the solvent for an hour. The solvent was allowed to evaporate to leave behind a mixture of crystalline products.  The product probably resulted from the hydrolysis of the phosphine.

### Refinement

The carbon-bound H-atoms were generated geometrically (C—H 0.95 Å) and were allowed to ride on their parent atoms, with *U*(H) fixed at 1.2*U*_eq_(C). The water H-atoms were located in a difference Fourier map, and were refined with a distance restraint of O–H 0.84±0.01 Å; temperature factors were freely refined.

### Crystal data

**Table:**

C_18_H_15_OP·0.5H_2_O	*F*(000) = 1208
*M**_r_* = 287.28	*D*_x_ = 1.287 Mg m^−^^3^
Monoclinic, *C**c*	Mo *K*α radiation, λ = 0.71073 Å
Hall symbol: C -2yc	Cell parameters from 9366 reflections
*a* = 9.4313 (1) Å	θ = 2.4–28.2°
*b* = 32.1930 (4) Å	µ = 0.18 mm^−^^1^
*c* = 10.8435 (1) Å	*T* = 100 K
β = 115.742 (1)°	Block, colorless
*V* = 2965.59 (6) Å^3^	0.30 × 0.25 × 0.20 mm
*Z* = 8	

### Data collection

**Table:**

Bruker SMART APEX diffractometer	6609 independent reflections
Radiation source: fine-focus sealed tube	6504 reflections with *I* > 2σ(*I*)
graphite	*R*_int_ = 0.019
ω scans	θ_max_ = 27.5°, θ_min_ = 2.1°
Absorption correction: multi-scan (*SADABS*; Sheldrick, 1996)	*h* = −12→12
*T*_min_ = 0.947, *T*_max_ = 0.965	*k* = −41→41
14206 measured reflections	*l* = −14→14

### Refinement

**Table:**

Refinement on *F*^2^	Secondary atom site location: difference Fourier map
Least-squares matrix: full	Hydrogen site location: inferred from neighbouring sites
*R*[*F*^2^ > 2σ(*F*^2^)] = 0.026	H atoms treated by a mixture of independent and constrained refinement
*wR*(*F*^2^) = 0.065	*w* = 1/[σ^2^(*F*_o_^2^) + (0.0453*P*)^2^] where *P* = (*F*_o_^2^ + 2*F*_c_^2^)/3
*S* = 1.02	(Δ/σ)_max_ = 0.001
6609 reflections	Δρ_max_ = 0.24 e Å^−^^3^
379 parameters	Δρ_min_ = −0.17 e Å^−^^3^
4 restraints	Absolute structure: Flack (1983), 3203 Friedel pairs
Primary atom site location: structure-invariant direct methods	Flack parameter: 0.02 (5)

### Fractional atomic coordinates and isotropic or equivalent isotropic displacement parameters (Å^2^)

**Table:**

	*x*	*y*	*z*	*U*_iso_*/*U*_eq_	
P1	0.50187 (5)	0.437805 (13)	0.50006 (5)	0.01863 (12)	
P2	0.27142 (5)	0.309772 (14)	−0.01509 (4)	0.01939 (12)	
O1	0.39737 (18)	0.40302 (4)	0.42266 (15)	0.0290 (3)	
O2	0.2633 (2)	0.34385 (4)	0.07577 (16)	0.0313 (3)	
O1W	0.1024 (2)	0.37124 (8)	0.2330 (2)	0.0539 (5)	
H1	0.189 (3)	0.3741 (11)	0.301 (3)	0.090 (14)*	
H2	0.126 (4)	0.3598 (9)	0.176 (3)	0.062 (10)*	
C1	0.6506 (2)	0.44805 (6)	0.4419 (2)	0.0233 (4)	
C2	0.6694 (3)	0.41888 (7)	0.3542 (2)	0.0324 (5)	
H2A	0.6044	0.3949	0.3272	0.039*	
C3	0.7822 (3)	0.42496 (8)	0.3069 (3)	0.0454 (7)	
H3	0.7941	0.4054	0.2466	0.055*	
C4	0.8776 (3)	0.45964 (9)	0.3476 (3)	0.0485 (7)	
H4	0.9556	0.4636	0.3154	0.058*	
C5	0.8609 (3)	0.48886 (8)	0.4353 (3)	0.0407 (6)	
H5	0.9275	0.5125	0.4633	0.049*	
C6	0.7458 (2)	0.48303 (7)	0.4817 (2)	0.0303 (5)	
H6	0.7325	0.5030	0.5405	0.036*	
C7	0.3943 (2)	0.48571 (5)	0.4777 (2)	0.0211 (4)	
C8	0.2742 (2)	0.49264 (7)	0.3473 (2)	0.0281 (5)	
H8	0.2488	0.4719	0.2787	0.034*	
C9	0.1909 (3)	0.53010 (7)	0.3171 (3)	0.0344 (5)	
H9	0.1107	0.5352	0.2277	0.041*	
C10	0.2272 (3)	0.55987 (6)	0.4198 (3)	0.0334 (6)	
H10	0.1716	0.5855	0.4000	0.040*	
C11	0.3420 (3)	0.55246 (7)	0.5488 (3)	0.0313 (5)	
H11	0.3636	0.5727	0.6183	0.038*	
C12	0.4276 (3)	0.51563 (6)	0.5793 (2)	0.0263 (4)	
H12	0.5081	0.5109	0.6689	0.032*	
C13	0.6082 (2)	0.42669 (6)	0.6805 (2)	0.0189 (4)	
C14	0.5294 (2)	0.42678 (6)	0.7640 (2)	0.0245 (4)	
H14	0.4233	0.4360	0.7282	0.029*	
C15	0.6055 (3)	0.41348 (7)	0.8986 (2)	0.0283 (4)	
H15	0.5518	0.4139	0.9551	0.034*	
C16	0.7600 (3)	0.39947 (7)	0.9512 (2)	0.0303 (5)	
H16	0.8118	0.3903	1.0434	0.036*	
C17	0.8380 (2)	0.39900 (7)	0.8685 (2)	0.0280 (5)	
H17	0.9434	0.3892	0.9045	0.034*	
C18	0.7640 (2)	0.41266 (6)	0.7337 (2)	0.0238 (4)	
H18	0.8188	0.4125	0.6780	0.029*	
C19	0.4727 (2)	0.29581 (6)	0.02612 (19)	0.0229 (4)	
C20	0.5931 (3)	0.32190 (7)	0.1102 (2)	0.0350 (5)	
H20	0.5702	0.3463	0.1476	0.042*	
C21	0.7491 (3)	0.31176 (9)	0.1393 (3)	0.0490 (7)	
H21	0.8326	0.3293	0.1971	0.059*	
C22	0.7812 (3)	0.27687 (9)	0.0848 (3)	0.0532 (8)	
H22	0.8873	0.2706	0.1044	0.064*	
C23	0.6632 (3)	0.25051 (8)	0.0020 (3)	0.0454 (6)	
H23	0.6875	0.2261	−0.0341	0.054*	
C24	0.5076 (2)	0.26023 (6)	−0.0279 (2)	0.0294 (5)	
H24	0.4250	0.2424	−0.0855	0.035*	
C25	0.1779 (2)	0.26268 (5)	0.0024 (2)	0.0205 (3)	
C26	0.1924 (2)	0.25324 (6)	0.1333 (2)	0.0248 (4)	
H26	0.2439	0.2721	0.2068	0.030*	
C27	0.1310 (2)	0.21616 (6)	0.1556 (2)	0.0288 (4)	
H27	0.1419	0.2096	0.2448	0.035*	
C28	0.0546 (3)	0.18885 (6)	0.0493 (3)	0.0288 (5)	
H28	0.0132	0.1636	0.0656	0.035*	
C29	0.0377 (2)	0.19808 (6)	−0.0821 (2)	0.0249 (4)	
H29	−0.0167	0.1794	−0.1555	0.030*	
C30	0.1007 (2)	0.23465 (6)	−0.1051 (2)	0.0230 (4)	
H30	0.0915	0.2407	−0.1941	0.028*	
C31	0.1855 (2)	0.32405 (6)	−0.1932 (2)	0.0202 (4)	
C32	0.0223 (2)	0.32522 (6)	−0.2683 (2)	0.0245 (4)	
H32	−0.0436	0.3158	−0.2282	0.029*	
C33	−0.0444 (3)	0.34019 (7)	−0.4020 (3)	0.0297 (5)	
H33	−0.1555	0.3407	−0.4534	0.036*	
C34	0.0521 (3)	0.35441 (7)	−0.4601 (2)	0.0283 (4)	
H34	0.0063	0.3647	−0.5512	0.034*	
C35	0.2138 (2)	0.35372 (6)	−0.3869 (2)	0.0249 (4)	
H35	0.2791	0.3636	−0.4270	0.030*	
C36	0.2802 (2)	0.33844 (6)	−0.2535 (2)	0.0225 (4)	
H36	0.3915	0.3378	−0.2028	0.027*	

### Atomic displacement parameters (Å^2^)

**Table:**

	*U*^11^	*U*^22^	*U*^33^	*U*^12^	*U*^13^	*U*^23^
P1	0.0212 (3)	0.0158 (2)	0.0184 (2)	0.00008 (17)	0.00812 (19)	−0.00098 (18)
P2	0.0238 (3)	0.0167 (2)	0.0194 (3)	−0.00428 (18)	0.0110 (2)	−0.00323 (18)
O1	0.0326 (8)	0.0215 (6)	0.0255 (7)	−0.0022 (6)	0.0056 (6)	−0.0038 (6)
O2	0.0468 (9)	0.0238 (7)	0.0302 (8)	−0.0088 (6)	0.0232 (7)	−0.0084 (6)
O1W	0.0235 (9)	0.0977 (15)	0.0408 (9)	−0.0137 (10)	0.0144 (9)	−0.0238 (10)
C1	0.0262 (9)	0.0265 (9)	0.0214 (9)	0.0102 (7)	0.0143 (8)	0.0097 (7)
C2	0.0457 (13)	0.0306 (10)	0.0247 (10)	0.0165 (10)	0.0188 (10)	0.0090 (8)
C3	0.0653 (17)	0.0488 (15)	0.0371 (13)	0.0314 (13)	0.0361 (13)	0.0198 (12)
C4	0.0477 (15)	0.0674 (17)	0.0475 (15)	0.0333 (14)	0.0365 (13)	0.0341 (14)
C5	0.0314 (11)	0.0453 (13)	0.0510 (15)	0.0066 (10)	0.0231 (11)	0.0226 (12)
C6	0.0300 (12)	0.0303 (10)	0.0333 (11)	0.0069 (8)	0.0162 (10)	0.0107 (9)
C7	0.0211 (9)	0.0156 (8)	0.0291 (10)	0.0009 (6)	0.0131 (9)	0.0010 (8)
C8	0.0241 (10)	0.0280 (10)	0.0312 (11)	0.0055 (8)	0.0111 (9)	0.0009 (8)
C9	0.0243 (10)	0.0346 (11)	0.0415 (13)	0.0065 (9)	0.0115 (10)	0.0091 (10)
C10	0.0275 (12)	0.0197 (10)	0.0591 (16)	0.0041 (8)	0.0244 (11)	0.0075 (9)
C11	0.0319 (11)	0.0197 (9)	0.0502 (14)	−0.0025 (8)	0.0251 (11)	−0.0055 (9)
C12	0.0299 (10)	0.0210 (9)	0.0299 (11)	−0.0030 (8)	0.0148 (9)	−0.0021 (8)
C13	0.0222 (9)	0.0155 (8)	0.0180 (9)	−0.0035 (7)	0.0076 (8)	−0.0010 (7)
C14	0.0238 (9)	0.0262 (10)	0.0264 (10)	0.0004 (7)	0.0135 (8)	−0.0015 (8)
C15	0.0323 (11)	0.0327 (11)	0.0280 (10)	−0.0014 (9)	0.0208 (9)	0.0013 (9)
C16	0.0342 (11)	0.0332 (10)	0.0226 (10)	−0.0014 (9)	0.0116 (9)	0.0028 (8)
C17	0.0187 (9)	0.0354 (11)	0.0277 (11)	−0.0024 (8)	0.0080 (8)	0.0028 (9)
C18	0.0232 (9)	0.0280 (10)	0.0240 (10)	0.0002 (8)	0.0138 (8)	0.0039 (8)
C19	0.0184 (8)	0.0265 (9)	0.0196 (9)	−0.0039 (7)	0.0045 (7)	0.0076 (7)
C20	0.0317 (12)	0.0351 (11)	0.0275 (11)	−0.0132 (9)	0.0031 (10)	0.0088 (9)
C21	0.0240 (11)	0.0537 (16)	0.0466 (15)	−0.0151 (11)	−0.0058 (11)	0.0241 (12)
C22	0.0243 (11)	0.0564 (16)	0.0723 (19)	0.0065 (11)	0.0148 (12)	0.0419 (15)
C23	0.0337 (12)	0.0396 (12)	0.0683 (17)	0.0134 (10)	0.0273 (13)	0.0277 (13)
C24	0.0249 (10)	0.0292 (10)	0.0341 (12)	0.0042 (8)	0.0129 (9)	0.0092 (8)
C25	0.0193 (9)	0.0191 (8)	0.0232 (9)	−0.0012 (7)	0.0094 (8)	−0.0006 (8)
C26	0.0221 (9)	0.0283 (10)	0.0254 (10)	−0.0014 (8)	0.0117 (8)	0.0001 (8)
C27	0.0290 (10)	0.0292 (10)	0.0339 (11)	−0.0009 (9)	0.0191 (9)	0.0036 (9)
C28	0.0217 (10)	0.0204 (10)	0.0475 (14)	0.0015 (7)	0.0179 (9)	0.0040 (8)
C29	0.0204 (9)	0.0186 (9)	0.0322 (11)	0.0001 (7)	0.0082 (8)	−0.0022 (8)
C30	0.0222 (9)	0.0203 (9)	0.0245 (10)	0.0034 (7)	0.0082 (8)	−0.0010 (8)
C31	0.0218 (9)	0.0156 (8)	0.0213 (10)	0.0012 (7)	0.0076 (8)	−0.0012 (8)
C32	0.0207 (9)	0.0230 (10)	0.0314 (11)	−0.0001 (7)	0.0128 (8)	−0.0013 (8)
C33	0.0195 (9)	0.0297 (10)	0.0326 (11)	0.0015 (8)	0.0045 (9)	0.0017 (9)
C34	0.0304 (10)	0.0293 (10)	0.0214 (10)	0.0038 (8)	0.0076 (9)	0.0057 (8)
C35	0.0269 (10)	0.0268 (9)	0.0234 (10)	0.0010 (8)	0.0131 (9)	0.0035 (8)
C36	0.0191 (9)	0.0235 (9)	0.0246 (10)	−0.0007 (7)	0.0093 (8)	−0.0011 (8)

### Geometric parameters (Å, °)

**Table:**

P1—O1	1.4871 (15)	C16—C17	1.385 (3)
P1—C1	1.801 (2)	C16—H16	0.9500
P1—C7	1.8035 (18)	C17—C18	1.390 (3)
P1—C13	1.805 (2)	C17—H17	0.9500
P2—O2	1.4985 (14)	C18—H18	0.9500
P2—C31	1.799 (2)	C19—C20	1.388 (3)
P2—C25	1.8051 (19)	C19—C24	1.390 (3)
P2—C19	1.8076 (19)	C20—C21	1.402 (4)
O1W—H1	0.84 (3)	C20—H20	0.9500
O1W—H2	0.83 (3)	C21—C22	1.363 (4)
C1—C6	1.387 (3)	C21—H21	0.9500
C1—C2	1.401 (3)	C22—C23	1.377 (4)
C2—C3	1.381 (4)	C22—H22	0.9500
C2—H2A	0.9500	C23—C24	1.394 (3)
C3—C4	1.381 (4)	C23—H23	0.9500
C3—H3	0.9500	C24—H24	0.9500
C4—C5	1.395 (4)	C25—C26	1.399 (3)
C4—H4	0.9500	C25—C30	1.402 (3)
C5—C6	1.394 (3)	C26—C27	1.393 (3)
C5—H5	0.9500	C26—H26	0.9500
C6—H6	0.9500	C27—C28	1.378 (3)
C7—C12	1.393 (3)	C27—H27	0.9500
C7—C8	1.394 (3)	C28—C29	1.395 (3)
C8—C9	1.398 (3)	C28—H28	0.9500
C8—H8	0.9500	C29—C30	1.389 (3)
C9—C10	1.395 (4)	C29—H29	0.9500
C9—H9	0.9500	C30—H30	0.9500
C10—C11	1.367 (4)	C31—C32	1.395 (3)
C10—H10	0.9500	C31—C36	1.395 (3)
C11—C12	1.391 (3)	C32—C33	1.392 (3)
C11—H11	0.9500	C32—H32	0.9500
C12—H12	0.9500	C33—C34	1.390 (3)
C13—C14	1.398 (3)	C33—H33	0.9500
C13—C18	1.400 (3)	C34—C35	1.379 (3)
C14—C15	1.385 (3)	C34—H34	0.9500
C14—H14	0.9500	C35—C36	1.393 (3)
C15—C16	1.389 (3)	C35—H35	0.9500
C15—H15	0.9500	C36—H36	0.9500
			
O1—P1—C1	111.49 (10)	C16—C17—C18	120.80 (19)
O1—P1—C7	111.83 (9)	C16—C17—H17	119.6
C1—P1—C7	105.96 (9)	C18—C17—H17	119.6
O1—P1—C13	112.48 (9)	C17—C18—C13	119.51 (18)
C1—P1—C13	105.40 (9)	C17—C18—H18	120.2
C7—P1—C13	109.28 (9)	C13—C18—H18	120.2
O2—P2—C31	113.00 (9)	C20—C19—C24	119.9 (2)
O2—P2—C25	112.26 (9)	C20—C19—P2	118.93 (17)
C31—P2—C25	108.33 (9)	C24—C19—P2	121.10 (15)
O2—P2—C19	111.57 (10)	C19—C20—C21	119.1 (2)
C31—P2—C19	105.30 (9)	C19—C20—H20	120.5
C25—P2—C19	105.90 (9)	C21—C20—H20	120.5
H1—O1W—H2	104 (3)	C22—C21—C20	120.2 (2)
C6—C1—C2	120.0 (2)	C22—C21—H21	119.9
C6—C1—P1	122.44 (15)	C20—C21—H21	119.9
C2—C1—P1	117.53 (17)	C21—C22—C23	121.4 (2)
C3—C2—C1	120.1 (2)	C21—C22—H22	119.3
C3—C2—H2A	119.9	C23—C22—H22	119.3
C1—C2—H2A	119.9	C22—C23—C24	119.0 (3)
C4—C3—C2	119.8 (2)	C22—C23—H23	120.5
C4—C3—H3	120.1	C24—C23—H23	120.5
C2—C3—H3	120.1	C19—C24—C23	120.4 (2)
C3—C4—C5	120.8 (2)	C19—C24—H24	119.8
C3—C4—H4	119.6	C23—C24—H24	119.8
C5—C4—H4	119.6	C26—C25—C30	119.38 (18)
C6—C5—C4	119.5 (2)	C26—C25—P2	116.88 (15)
C6—C5—H5	120.3	C30—C25—P2	123.64 (16)
C4—C5—H5	120.3	C27—C26—C25	119.83 (19)
C1—C6—C5	119.8 (2)	C27—C26—H26	120.1
C1—C6—H6	120.1	C25—C26—H26	120.1
C5—C6—H6	120.1	C28—C27—C26	120.5 (2)
C12—C7—C8	119.78 (18)	C28—C27—H27	119.8
C12—C7—P1	124.40 (16)	C26—C27—H27	119.8
C8—C7—P1	115.79 (16)	C27—C28—C29	120.29 (19)
C7—C8—C9	120.1 (2)	C27—C28—H28	119.9
C7—C8—H8	119.9	C29—C28—H28	119.9
C9—C8—H8	119.9	C30—C29—C28	119.7 (2)
C10—C9—C8	119.2 (2)	C30—C29—H29	120.1
C10—C9—H9	120.4	C28—C29—H29	120.1
C8—C9—H9	120.4	C29—C30—C25	120.2 (2)
C11—C10—C9	120.5 (2)	C29—C30—H30	119.9
C11—C10—H10	119.8	C25—C30—H30	119.9
C9—C10—H10	119.8	C32—C31—C36	119.09 (18)
C10—C11—C12	120.8 (2)	C32—C31—P2	120.15 (16)
C10—C11—H11	119.6	C36—C31—P2	120.39 (15)
C12—C11—H11	119.6	C33—C32—C31	120.15 (19)
C11—C12—C7	119.5 (2)	C33—C32—H32	119.9
C11—C12—H12	120.2	C31—C32—H32	119.9
C7—C12—H12	120.2	C34—C33—C32	119.85 (19)
C14—C13—C18	119.49 (18)	C34—C33—H33	120.1
C14—C13—P1	120.00 (15)	C32—C33—H33	120.1
C18—C13—P1	119.98 (15)	C35—C34—C33	120.7 (2)
C15—C14—C13	120.22 (18)	C35—C34—H34	119.6
C15—C14—H14	119.9	C33—C34—H34	119.6
C13—C14—H14	119.9	C34—C35—C36	119.32 (19)
C14—C15—C16	120.29 (19)	C34—C35—H35	120.3
C14—C15—H15	119.9	C36—C35—H35	120.3
C16—C15—H15	119.9	C35—C36—C31	120.85 (18)
C17—C16—C15	119.7 (2)	C35—C36—H36	119.6
C17—C16—H16	120.2	C31—C36—H36	119.6
C15—C16—H16	120.2		
			
O1—P1—C1—C6	169.22 (16)	O2—P2—C19—C20	−13.47 (18)
C7—P1—C1—C6	47.3 (2)	C31—P2—C19—C20	109.47 (17)
C13—P1—C1—C6	−68.45 (18)	C25—P2—C19—C20	−135.89 (16)
O1—P1—C1—C2	−11.38 (19)	O2—P2—C19—C24	168.31 (15)
C7—P1—C1—C2	−133.27 (16)	C31—P2—C19—C24	−68.75 (17)
C13—P1—C1—C2	110.95 (17)	C25—P2—C19—C24	45.89 (19)
C6—C1—C2—C3	−0.2 (3)	C24—C19—C20—C21	0.0 (3)
P1—C1—C2—C3	−179.65 (16)	P2—C19—C20—C21	−178.23 (18)
C1—C2—C3—C4	0.8 (3)	C19—C20—C21—C22	0.4 (3)
C2—C3—C4—C5	−0.4 (4)	C20—C21—C22—C23	−0.9 (4)
C3—C4—C5—C6	−0.4 (3)	C21—C22—C23—C24	0.9 (4)
C2—C1—C6—C5	−0.6 (3)	C20—C19—C24—C23	0.1 (3)
P1—C1—C6—C5	178.78 (16)	P2—C19—C24—C23	178.29 (18)
C4—C5—C6—C1	0.9 (3)	C22—C23—C24—C19	−0.6 (4)
O1—P1—C7—C12	144.49 (16)	O2—P2—C25—C26	−35.86 (18)
C1—P1—C7—C12	−93.83 (18)	C31—P2—C25—C26	−161.33 (15)
C13—P1—C7—C12	19.28 (19)	C19—P2—C25—C26	86.12 (16)
O1—P1—C7—C8	−37.70 (19)	O2—P2—C25—C30	147.75 (16)
C1—P1—C7—C8	83.97 (17)	C31—P2—C25—C30	22.27 (18)
C13—P1—C7—C8	−162.91 (15)	C19—P2—C25—C30	−90.28 (17)
C12—C7—C8—C9	2.2 (3)	C30—C25—C26—C27	0.3 (3)
P1—C7—C8—C9	−175.69 (16)	P2—C25—C26—C27	−176.21 (15)
C7—C8—C9—C10	−1.5 (3)	C25—C26—C27—C28	−0.7 (3)
C8—C9—C10—C11	−0.4 (3)	C26—C27—C28—C29	0.0 (3)
C9—C10—C11—C12	1.6 (3)	C27—C28—C29—C30	1.1 (3)
C10—C11—C12—C7	−0.9 (3)	C28—C29—C30—C25	−1.5 (3)
C8—C7—C12—C11	−1.0 (3)	C26—C25—C30—C29	0.7 (3)
P1—C7—C12—C11	176.70 (16)	P2—C25—C30—C29	177.05 (15)
O1—P1—C13—C14	−73.10 (19)	O2—P2—C31—C32	−76.16 (18)
C1—P1—C13—C14	165.21 (16)	C25—P2—C31—C32	48.88 (18)
C7—P1—C13—C14	51.73 (19)	C19—P2—C31—C32	161.83 (16)
O1—P1—C13—C18	98.50 (17)	O2—P2—C31—C36	96.75 (17)
C1—P1—C13—C18	−23.18 (19)	C25—P2—C31—C36	−138.21 (16)
C7—P1—C13—C18	−136.67 (16)	C19—P2—C31—C36	−25.27 (18)
C18—C13—C14—C15	0.6 (3)	C36—C31—C32—C33	0.6 (3)
P1—C13—C14—C15	172.26 (16)	P2—C31—C32—C33	173.60 (16)
C13—C14—C15—C16	−0.8 (3)	C31—C32—C33—C34	−0.7 (3)
C14—C15—C16—C17	0.2 (3)	C32—C33—C34—C35	0.2 (3)
C15—C16—C17—C18	0.5 (3)	C33—C34—C35—C36	0.2 (3)
C16—C17—C18—C13	−0.6 (3)	C34—C35—C36—C31	−0.3 (3)
C14—C13—C18—C17	0.1 (3)	C32—C31—C36—C35	−0.1 (3)
P1—C13—C18—C17	−171.57 (16)	P2—C31—C36—C35	−173.12 (15)

### Hydrogen-bond geometry (Å, °)

**Table:**

*D*—H···*A*	*D*—H	H···*A*	*D*···*A*	*D*—H···*A*
O1w—H1···O1	0.84 (3)	2.05 (2)	2.836 (2)	156 (4)
O1w—H2···O2	0.83 (3)	2.09 (2)	2.870 (2)	158 (3)
